# An Updated Review on Electrochemical Nanobiosensors for Neurotransmitter Detection

**DOI:** 10.3390/bios13090892

**Published:** 2023-09-19

**Authors:** Hye Kyu Choi, Jin-Ha Choi, Jinho Yoon

**Affiliations:** 1 Department of Chemistry and Chemical Biology, Rutgers, The State University of New Jersey, Piscataway, NJ 08854, USA; hye.choi@rutgers.edu; 2 School of Chemical Engineering, Clean Energy Research Center, Jeonbuk National University, Jeonju 54896, Republic of Korea; 3 Department of Biomedical-Chemical Engineering, The Catholic University of Korea, Bucheon 14662, Republic of Korea; 4 Department of Biotechnology, The Catholic University of Korea, Bucheon 14662, Republic of Korea

**Keywords:** electrochemistry, neurotransmitter, nanomaterials, neurological diseases, nanobiosensors

## Abstract

Neurotransmitters are chemical compounds released by nerve cells, including neurons, astrocytes, and oligodendrocytes, that play an essential role in the transmission of signals in living organisms, particularly in the central nervous system, and they also perform roles in realizing the function and maintaining the state of each organ in the body. The dysregulation of neurotransmitters can cause neurological disorders. This highlights the significance of precise neurotransmitter monitoring to allow early diagnosis and treatment. This review provides a complete multidisciplinary examination of electrochemical biosensors integrating nanomaterials and nanotechnologies in order to achieve the accurate detection and monitoring of neurotransmitters. We introduce extensively researched neurotransmitters and their respective functions in biological beings. Subsequently, electrochemical biosensors are classified based on methodologies employed for direct detection, encompassing the recently documented cell-based electrochemical monitoring systems. These methods involve the detection of neurotransmitters in neuronal cells in vitro, the identification of neurotransmitters emitted by stem cells, and the in vivo monitoring of neurotransmitters. The incorporation of nanomaterials and nanotechnologies into electrochemical biosensors has the potential to assist in the timely detection and management of neurological disorders. This study provides significant insights for researchers and clinicians regarding precise neurotransmitter monitoring and its implications regarding numerous biological applications.

## 1. Introduction

Neurotransmitters are chemical substances secreted from nerve cells. They serve as transporters, and play an important role in transmitting signals within nerves in living organisms, including the central nervous system (CNS), and in realizing the function and maintaining the state of each organ. A number of neurotransmitters, including dopamine and glutamate, play many roles in living organisms [1,2]. Additionally, these neurotransmitters contribute to the maturation and development of the brains of living organisms [3]. When a problem occurs in the production and transmission of neurotransmitters, it can potentially have fatal consequences in the signal transmission process through nerves, and may cause cranial nerve-related diseases [4,5]. Therefore, from a biomedical perspective, the accurate monitoring of neurotransmitter levels in the nervous system is required for the early prediction and treatment of cranial diseases, such as Parkinson’s, Alzheimer’s, and Huntington’s [6,7]. In addition, other recent reports have demonstrated that these neurotransmitters are associated with cancer occurrence [8]. Furthermore, the precise monitoring of neurotransmitter levels is advantageous, in that it is capable of providing a strategy for stem cell therapy, because it can be used to accurately monitor the differentiation stages of neural stem cells [9,10].

From this point of view, various biosensors based on fluorescence, Raman spectroscopy, and electrochemical techniques have been developed for the accurate monitoring of neurotransmitters [11,12,13]. Among these biosensing techniques, electrochemical biosensors have various advantages, including (among others) rapid response, sensitivity, selectivity, and multiple detection properties [14]. In the case of fluorescence analysis, invasive cell staining processes are inevitably required, and expert operational skills are generally necessary to conduct Raman spectroscopic analysis for the monitoring of neurotransmitters. Additionally, in contrast to other techniques, electrochemical biosensors can be developed based on different electrochemical techniques, such as cyclic voltammetry (CV), differential pulse voltammetry (DPV), and amperometry techniques. Furthermore, electrochemical biosensors can utilize various biomolecules, such as enzymes, proteins, and nucleic acids to detect numerous different target molecules, including small chemicals, nucleic acids (microribonucleic acid (miRNA), short interfering RNA (siRNA), viral deoxyribonucleic acid (DNA)/RNA, etc.), and neurotransmitters [15,16]. Moreover, using electrochemical techniques, noninvasive monitoring of cellular states based on the extracellular redox reactions becomes possible [17,18]. This noninvasive monitoring approach can be used directly for the development of neuronal stem cell therapy products by precisely monitoring the differentiation of neural stem cells, which is difficult to perform by using other types of technique.

However, biomolecule-assisted electrochemical biosensors for neurotransmitter detection are limited in terms of sensitivity and selectivity, owing to the low redox reaction efficiency and increased noise response. To address these problems, nanomaterials and nanotechnologies are frequently incorporated into electrochemical biosensors to improve their sensitivity and selectivity characteristics via (among others) the provision of large surface areas, an excellent template, and enhanced electron transfer [19,20]. Based on these studies, biosensors—which can accurately detect neurotransmitter changes without cells—or highly sensitive electrochemical biosensors—which can directly measure neurotransmitters released from cells—are being developed. Therefore, approaches that promote the comprehensive understanding and the development of biosensors capable of monitoring neurotransmitters based on electrochemical technology are necessary for early diagnosis and treatment of neurological diseases and the development of neural stem cell therapeutics.

**Figure 1:**
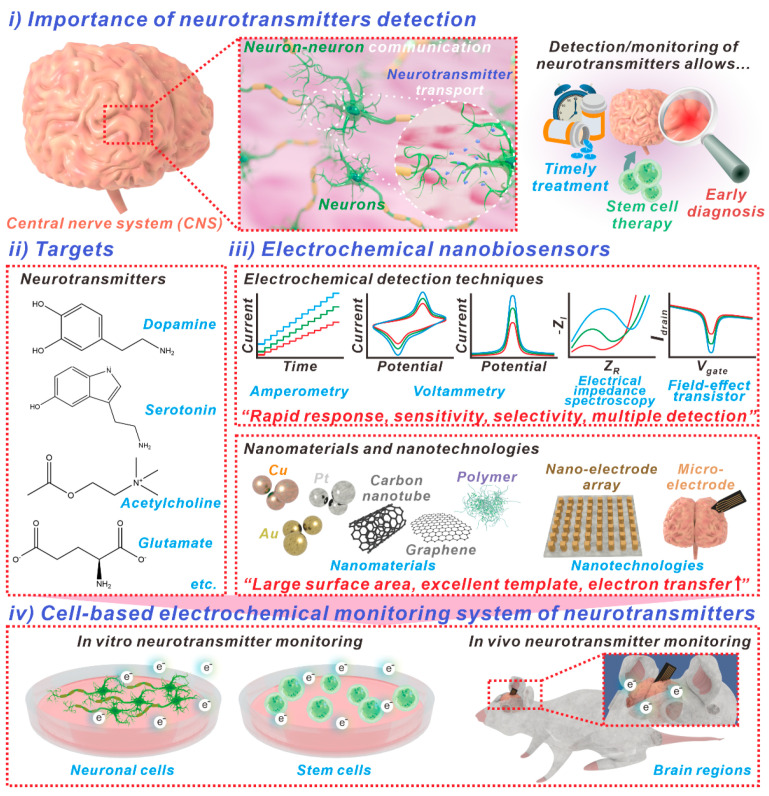
Schematic of electrochemical nanobiosensors used for neurotransmitter detection. (**i**) Detection of neurotransmitters is important, since a problem in the production and transmission of neurotransmitters could potentially have fatal consequences in the signal transmission process in nerves and may cause cranial nerve-related diseases. To achieve monitoring of (**ii**) the neurotransmitters sensitively and selectively, (**iii**) various nanomaterials and nanotechnologies have been applied in the development of electrochemical biosensors. Additionally, utilizing fabricated biosensors, the neurotransmitters in (**iv**) neuronal cells, stem cells, and animal models can be monitored.

In this review, we provide interdisciplinary information about electrochemical biosensors incorporating nanomaterials and nanotechnologies that enable the accurate detection or monitoring of neurotransmitters (Figure 1). First, extensively studied neurotransmitters that can be monitored electrochemically are presented, and their roles in living organisms are described. Subsequently, electrochemical biosensors that can detect neurotransmitters directly, regardless of living cells, are discussed; these biosensors are categorized on the basis of their respective electrochemical techniques in recently reported studies. Lastly, cell chips that are able to monitor the neurotransmitters derived from living cells are outlined; these chips can be categorized as either disease-related neurotransmitter detection biosensors or stem cell-related neurotransmitter detection biosensors.

## 2. Neurotransmitters for Early Diagnosis and Pathophysiological Monitoring

Neurotransmitters play a role as signal transmitters among connected nerve cells, mediate the occurrence of various diseases, and maintain a healthy body state [21,22]. Numerous neurotransmitters in the forms of monoamines, amino acids, peptides, purines, or gasotransmitters (a gas neurotransmitter form) are being studied as detection targets.

**Figure 2:**
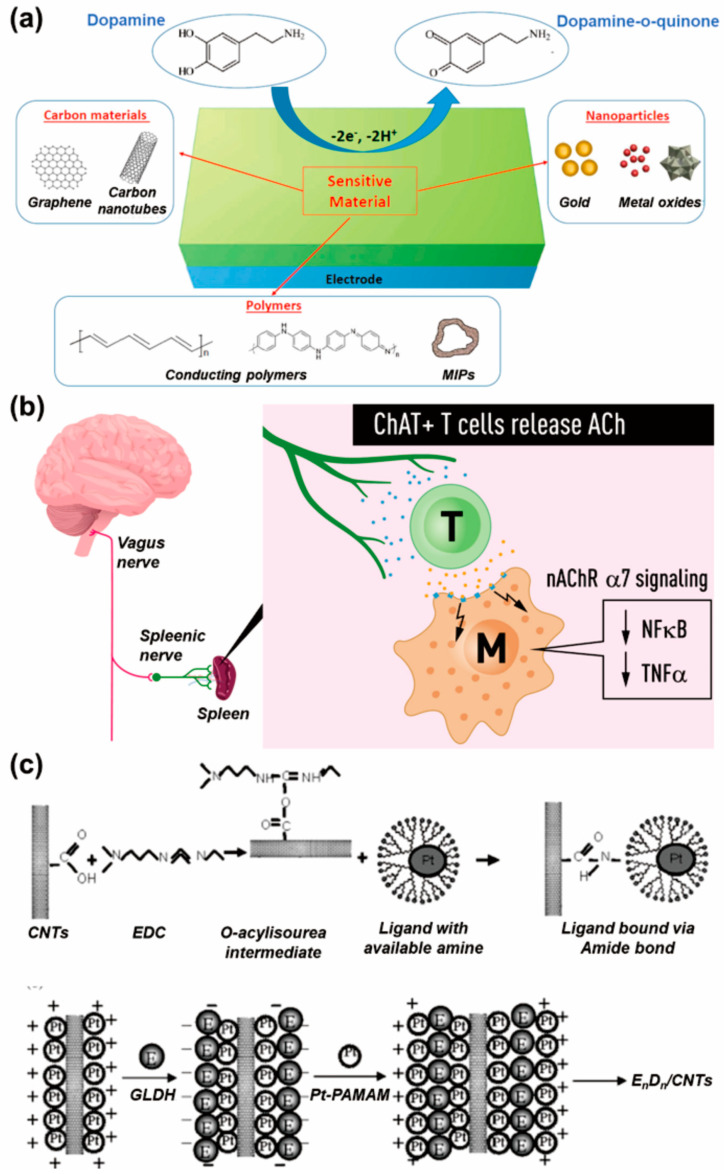
Representative neurotransmitters and their detection for early diagnosis and pathophysiological monitoring. (**a**) Electrochemical dopamine detection achieved via the conversion between dopamine and dopamine-o-quinone (reprinted with permission from [26]; Copyright © 2021 by the authors. Licensee: MDPI). (**b**) Role of acetylcholine in the immunomodulation by T cells (reprinted with permission from [30]; Copyright © 2019, The Association for the Publication of the Journal of Internal Medicine). (**c**) Electrochemical glutamate biosensor composed of glutamate dehydrogenase (GLDH), poly(amidoamine) dendrimer-encapsulated platinum nanoparticles (Pt-PAMAM) and carbon nanotubes (CNTs) (reprinted with permission from [37]; Copyright © 2007, Elsevier B.V.).

First, among the monoamine-type neurotransmitters, dopamine is one of the most extensively researched neurotransmitters. Dopamine is normally found in the CNS of living organisms and is one of the catecholamine families that has several important functions in the body, including motor control, motivation, and reinforcement, with important functional associations with the immune system [23]. The dopamine can be used in enzymatic reactions such as the reaction with dopamine ß-hydroxylase (DBH), or it can be electrochemically reacted with novel metals such as gold and silver. As a result of these reactivities of dopamine, it has been extensively studied as the target of electrochemical neurotransmitter biosensors [24,25]. Specifically, the nondestructive monitoring of dopaminergic cell differentiation is possible via the direct electrochemical reaction of dopamine, or through the use of an aptamer (which induces a redox function capable of reacting with dopamine), thus allowing the monitoring of cell-derived dopamine by switching between dopamine and dopamine-o-quinone (Figure 2a) [26]. Additional dopamine roles have recently become known, such as the regulation of inflammation and the activation of T-cells, thus establishing it as the most attractive neurotransmitter in the biosensor field [27]. Another well-known neurotransmitter in the form of a monoamine is serotonin. Serotonin, normally found in platelets and the CNS, is related to various physiological functions, such as memory, motor control, and learning, and is involved in inflammation and immunity [28,29]. In addition to these, there is acetylcholine, which is a monoamine-type neurotransmitter that plays a role in learning, memory, and muscle movement. Furthermore, acetylcholine is related to vagus-nerve-induced T-cell immunomodulation (Figure 2b) [30]. Acetylcholine is suitable for developing biosensors because it can react with acetylcholinesterase in an enzymatic manner through the conversion of acetylcholine into choline and acetic acid [31]. For instance, in the presence of acetylcholine and water, an acetylcholinesterase can degrade the acetylcholine into choline and acetic acid, following which the produced acetic acid is further converted into acetate and hydrogen ions. The production of these ions via the detection of acetylcholine by acetylcholinesterase can be easily measured using electrochemical techniques [32]. In addition to this, other monoamine-type neurotransmitters, such as catecholamine and norepinephrine, are being studied as biosensor targets [33,34].

Among the neurotransmitters that take the form of amino acids, glutamate is being studied extensively. Glutamate is distributed extensively in brain tissue, and at a higher concentration than other amino acids, and performs an important role in neurotransmission in the CNS, involving cellular metabolic pathways such as the urea and Krebs cycles [35]. Using glutamate oxidase or glutamate dehydrogenase (GLDH), electrochemical detection of glutamate can be easily achieved; accordingly, this area has been extensively studied, and numerous glutamate electrochemical biosensors have been reported [36]. For example, GLDH was immobilized on the poly(amidoamine) dendrimer-encapsulated platinum nanoparticles and carbon nanotubes (CNTs) to achieve its detection in an electrochemical manner (Figure 2c) [37]. Additionally, there are other attractive amino-acid-type neurotransmitters, including glycine and γ-aminobutyric acid (GABA) [38,39]. As the peptide form of neurotransmitters, opioids, which are involved with the release of other neurotransmitters (including dopamine, acetylcholine, and norepinephrine) during neurotransmission in the CNS, have been extensively studied [40]. As such, the real-time monitoring of various neurotransmitters is very important from a biomedical point of view in order to diagnose neurological diseases and determine appropriate therapies in early disease stages. To achieve this goal, various nanomaterials and nanotechnologies are being used to improve the accuracy of electrochemical neurotransmitter biosensors.

## 3. Recent Advances in Electrochemical Nanobiosensors for Neurotransmitter Detection

The accurate monitoring of the aforementioned neurotransmitters is of primary importance in understanding the complexities of neural systems and diagnosing neurological disorders [41]. Detailed information on neurotransmitters is presented in Table 1. Over the years, diverse biosensors employing fluorescence, Raman spectroscopy, and electrochemical techniques have been developed for the detection and monitoring of neurotransmitters [42]. Among these methodologies, electrochemistry for biosensors has emerged as a highly promising and versatile technique, thus offering numerous advantages, such as rapid response, high sensitivity and selectivity, and multiple detection capability, for the development of robust biosensing platforms [43].

Despite the advantages of electrochemical biosensors for neurotransmitter detection, certain challenges may arise with respect to sensitivity and selectivity owing to limitations in redox reaction efficiency and high noise levels [44]. To address these limitations, researchers have focused on the incorporation of nanomaterials and nanotechnologies, aiming to enhance the sensitivity and selectivity of electrochemical biosensors [45,46]. Nanomaterials offer unique properties, such as the provision of large surface areas and excellent templating capabilities, which improve electron transfer and overall sensor performance. On the basis of of these efforts, researchers are actively developing electrochemical biosensors capable of accurately detecting neurotransmitters. The outcomes of these advancements are promising for the early diagnosis and targeted treatments of neurological disorders, as well as disease therapeutics. This chapter aims to introduce recent advances in electrochemical nanobiosensors for neurotransmitter detection, including the methodologies utilized in electrochemical nanobiosensors, such as amperometric, voltammetric, electrochemical impedance spectroscopy (EIS), and field-effect transistors (FET).

### 3.1. Amperometric Biosensors

**Figure 3:**
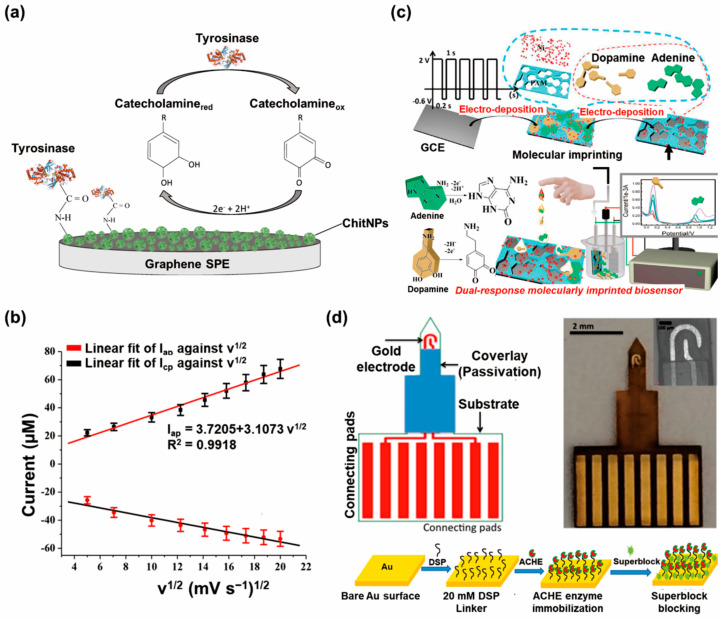
Recent types of electrochemical nanobiosensors used for neurotransmitter detection. (**a**) Structure of the amperometric biosensor for the detection of various catecholamines (reprinted with permission from [54]; Copyright © 2022 by the authors. Licensee: MDPI). (**b**) Plot of the peak current of the cyclic voltammogram of carbon quantum dot/copper oxide nanocomplex-modified glassy carbon electrode in 0.4 mM epinephrine solution as a function of the square root of scan rate (reprinted with permission from [64]; Copyright © 2022, Elsevier B.V.). (**c**) Schematic of the assembly process of the dual-response MIP-sensing membrane and electrochemical detection of dopamine and adenine (reprinted with permission from [73]; Copyright © 2022, Elsevier B.V.). (**d**) Schematic of the assembly process of the dual-response MIP-sensing membrane and electrochemical detection of dopamine and adenine (reprinted with permission from [78]; Copyright © 2023, Elsevier B.V.).

Amperometric biosensors represent a major innovation in the field of neurotransmitter detection, offering sensitive and selective analysis of these crucial signaling molecules. By harnessing electrochemical currents, amperometric biosensors can provide real-time measurements of neurotransmitter concentrations, thus making them invaluable tools for understanding neural processes and diagnosing neurological disorders [47]. Amperometric biosensors for neurotransmitter detection consist of three main components, namely, electrodes, biorecognition elements, and working solutions [48,49]. The biorecognition element is typically an enzyme that is selective to the neurotransmitter of interest. When a sample containing the neurotransmitter is introduced to the biosensor, the neurotransmitter interacts with the biorecognition element on the electrode’s surface [50]. This interaction triggers a specific enzymatic reaction in which the neurotransmitter is either oxidized or reduced. As a result of the enzymatic reaction, there is a change in the number of electrons on the electrode’s surface. This change in the number of electrons leads to the generation of an electrical current, which is directly related to the concentration of the neurotransmitter in the sample [51]. By calibrating the biosensor with known standards of the neurotransmitter, the measured current can be correlated with the actual concentration of the neurotransmitter in the sample. Additionally, through Faraday’s law, the number of electroactive molecules can be directly quantitatively counted. For example, open carbon nanopipettes (CNPs) have been employed to manipulate the translocation of vesicles of varying sizes using the orifices of the pipettes for the electrochemical detection of catecholamine molecules from the vesicles [52]. In this study, the number of catecholamine molecules was quantified on the basis of Faraday’s law, N_molecule_ = N_A_Q/nF. Here, N_A_, Q, n, and F are Avogadro’s number, the charge calculated by integrating the current over time, the number of electrons transferred during the catecholamine oxidation reaction (*n* = 2), and the Faraday constant, respectively. For example, dopamine was detected by utilizing an amperometric biosensor composed of a carbon dot 3-Chloropropyl-trimethoxysilane (CDs-CPTMS)-modified working electrode [53]. Herein, tyrosinase (which can oxidize dopamine to dopamine-o-quinone) was immobilized on the CDs-CPTMS-modified electrode. Based on the electrochemical reduction of dopamine-o-quinone at -0.15 V, the amount of dopamine was monitored in synthetic blood. Owing to the electrochemical properties of CDs-CPTMS, dopamine was sensitively and selectively detected with a limit of detection of 1.0 nM in a highly reproducible manner. Other amperometric biosensors (consisting of tyrosinase and chitosan nanoparticles) have been developed to detect the various catecholamines, such as dopamine, epinephrine, and norepinephrine (Figure 3a) [54]. The synthesized chitosan nanoparticles demonstrated highly enhanced immobilization of tyrosinase on the nanoparticles. To enhance the conductivity of the nanoparticles, graphene was incorporated into the nanocomposite matrix. The developed tyrosinase/chitosan nanoparticles/graphene nanocomplex-based amperometric biosensor showed improved sensitive detection of catecholamines, with a limit of detection of 0.17 µM, owing to its large surface area and excellent conductivity. In another research study, poly(3,4-ethylenedioxythiophene) (PEDOT)-based carbon electrodes were utilized for the detection of acetylcholine [55]. PEDOT is conductive polymer that is extensively used because of its high conductivity and stability. When a negative potential is applied, anionic analytes from tetrabutylammonium hexafluorophosphate encapsulated within the PEDOT film migrate into the poly(vinyl) chloride (PVC) membrane, leading to the reduction of the PEDOT film. This migration results in an accumulation of excess negative charge within the PVC membrane, thereby attracting aqueous cationic ions. As a consequence, cations like acetylcholine are detected. The specificity for acetylcholine detection over other cationic species is conferred by the presence of the selectively added ionophore in the PVC membrane. In this study, heptakis(2,3,6-tri-O-methyl)-β-cyclodextrin was employed as the ionophore in the PVC membrane, a choice that had previously demonstrated excellent selectivity for acetylcholine detection. Acetylcholine was more selectively detected than other interfering small molecules, such as serotonin, amino acids, and ascorbic acid. In one study, to enhance the sensitivity of amperometric biosensors for acetylcholine, platinum nanoparticles with porous graphene oxide nanosheets were applied [56]. Owing to the high conductivity of fabricated composites, sensitive and rapid detection of the acetylcholine was achieved, with a limit of detection of 1 nM. Moreover, the technique of single-cell amperometry (SCA) has been widely used in the field of exocytosis research, as demonstrated by several studies [57,58]. The direct detection of electrochemically active neurotransmitters, such as dopamine, at the single-cell level can be achieved by the utilization of micro or nanoelectrodes. This approach allows for the detection of neurotransmitters that are secreted from individual vesicles during the process of exocytosis. By conducting an analysis of the recorded current transients resulting from the oxidation process of neurotransmitters while a constant potential is applied to the electrode surface, it is possible to measure the quantity of neurotransmitters released during a single exocytotic event [59].

### 3.2. Voltammetric Biosensors

Voltammetric biosensors operate by applying a controlled potential to the working electrode, thus allowing the detection of electrochemical reactions involving neurotransmitters. In comparison to amperometric biosensors, voltammetric biosensors offer distinct advantages. One of the advantages of voltammetric biosensors compared with amperometric biosensors is their ability to investigate the electrochemical properties of neurotransmitters over a range of potentials [60]. While amperometry measures the constant current produced during an enzymatic reaction, voltammetry provides a more comprehensive view by examining how the current changes with variations in potential. This feature allows researchers to obtain deeper insights into the complex electrochemical behavior of neurotransmitters, thus leading to enhanced selectivity and sensitivity in their detection [26]. In the field of voltammetric biosensors, CV and DPV stand out.

#### 3.2.1. Cyclic Voltammetry

CV involves the sweeping of the potential back and forth, thus generating a cyclic current response that reveals the redox behavior of neurotransmitters. This cyclic response provides valuable information about the oxidation and reduction processes, thus enabling the precise quantitative analysis and identification of neurotransmitters [61]. The oxidation peak observed in a voltammogram is a result of the oxidation process occurring at the electrode surface, specifically involving the neurotransmitters. It is noteworthy that the amplitude of this oxidation peak is directly proportional to the concentration of the existing neurotransmitters. Consequently, the quantification of neurotransmitter concentration can be achieved by directly measuring the peak current values. Given the aforementioned criteria, the utilization of CV has been investigated as a viable approach for the detection of electroactive neurotransmitters [62]. For instance, a CV-based nanobiosensor composed of polypyrrole-derived carbon nanotubes was developed to detect dopamine [63]. In that research study, heteroatom-doped CNTs were synthesized based on the pyrolysis of polypyrrole nanotubes. A temperature of 800 ºC was used as an optimized synthesis temperature, with which the fabricated heteroatom-doped CNT-modified electrodes exhibited high conductivity and large surface area. Compared with polypyrrole, polypyrrole-derived carbon nanotubes produced at a temperature of 800 ºC showed higher electrochemical sensing performance. Based on the CV technique, dopamine in 0.1 M phosphate-buffered saline was detected using electrodes that exhibited a limit of detection of 0.2 µM. In another study, carbon quantum dots/copper oxide nanocomplexes were utilized to detect epinephrine (Figure 3b) [64]. Copper oxide nanoparticles are biocompatible, possess considerable surface areas, and have elevated electrical conductivity. Given the aforementioned characteristics of copper oxide nanoparticles and the established electrocatalytic capabilities of carbon quantum dots in the context of electrochemical oxidation of epinephrine, the incorporation of copper oxide nanoparticles and carbon quantum dots resulted in the formation of electrocatalytic structures for electro-oxidation of epinephrine that exhibited remarkable sensitivity, with a limit of detection (LOD) of 15.99 µM. The surface areas calculated for the bare electrode and the electrode with carbon quantum dot/copper oxide nanoparticles were 7.00 cm^2^ and 7.70 cm^2^, respectively. Additionally, the developed electrochemical biosensor demonstrated selective epinephrine detection in the presence of ascorbic acid, dopamine, and uric acid. Fast Scan Cyclic Voltammetry (FSCV) is a technique that has contributed to remarkable developments in the field of cyclic voltammetry, including in the investigation of dynamic electrochemical reactions. FSCV involves the rapid application of a triangular waveform potential to a working electrode, enabling real-time monitoring of fast, dynamic reactions [65]. Through the utilization of current measurements, researchers are able to acquire valuable information related to several biological processes, including, but not limited to, neurotransmitter release and uptake [66]. The utilization of this technique has demonstrated significant value in the field of neuroscience research, providing valuable insights into fundamental elements of brain functioning regarding addiction and several neurological disorders [67,68]. Moreover, FSCV has been utilized in several fields, such as environmental monitoring, medicinal research, and materials science, wherein the comprehension of fast electrochemical phenomena holds substantial importance [69,70].

#### 3.2.2. Differential Pulse Voltammetry

DPV, a variant of voltammetry, further augments sensitivity by utilizing short voltage pulses. This technique optimizes the signal-to-noise ratio, thus enabling voltammetric biosensors to detect even trace amounts of neurotransmitters with exceptional accuracy [71]. For instance, one study employed thermally reduced graphene oxide (rGO) to fabricate nanobiosensors for the detection of dopamine [72]. The thermal reduction of two distinct graphene oxide samples was conducted using malonic acid and P_2_O_5_ additives as precursors for the synthesis of carbon suboxide (C_3_O_2_). C_3_O_2_ plays a crucial role in repairing the conjugated aromatic structure of reduced graphene oxide sheets. In addition, the thermal treatment of graphene oxide in the presence of both malonic acid and the P_2_O_5_ mixture not only effectively restores the π-conjugated system of graphene layers, but also facilitates the successful incorporation of phosphorus (P) elements into the graphene-based material’s structure. The incorporation of P elements into the graphene layers creates topological defects and generates numerous active sites, which play a crucial role in enabling electron transfer to dopamine. While the bare electrode showed peak current values of 0.15 µA and −0.37 µA for I_pa_ and I_pc_, the developed biosensor showed values of 4.82 µA and −4.31 µA for I_pa_ and I_pc_. Utilizing the fabricated nanobiosensor, dopamine was successfully detected using the DPV technique with an LOD of 0.11 μM. In another study, the researchers took advantage of several key benefits offered by molecularly imprinted polymer (MIP) membranes prepared in situ (Figure 3c) [73]. These MIP membranes maintained their original morphology, exhibited strong adhesion to the collector, and allowed precise control over their structures. To fabricate a three-dimensional porous polyacrylamide–MIP matrix, they employed a single-step electropolymerization process of acrylamide monomers in the presence of nickel and template molecules directly on a glassy carbon electrode. The resulting Ni-polyacrylamide (PAM)–MIP matrix served as a remarkable sensor with a quantitative dual response toward dopamine and adenine, and successful detection was able to be conducted using DPV at the respective limit of detections of 0.12 µM and 0.15 µM. Additionally, compared with bare electrode, the fabricated MIP electrode showed substantially smaller charge resistance at the electrolyte/electrode interfaces. In both examples from the aforementioned studies, CV and DPV were utilized to detect the neurotransmitters. However, for the sensitive detection of neurotransmitters, DPV was the preferred method, owing to its higher sensitivity compared to CV. As a result, the researchers conducted neurotransmitter detection using DPV to achieve accurate and precise measurements, taking advantage of its enhanced sensitivity compared with CV.

### 3.3. Electrochemical Impedance Spectroscopy Biosensors

EIS has emerged as a very effective technique for the advancement of the field of nanobiosensors, exhibiting high sensitivity and selectivity in the field of neurotransmitter detection. EIS-based nanobiosensors take advantage of the dynamic changes in electrical impedance resulting from the specific binding events between neurotransmitters and biomolecular recognition elements immobilized on nanomaterial surfaces [74]. For example, anti-GABA antibodies were modified on a gold electrode to detect GABA [75]. Through the antibody–antigen reaction, GABA was captured on the gold substrate. The changed impedance parameters indicated the underlying biomolecular changes on the gold surface of the immunosensor. In another study, an aptamer-modified microelectrode was developed to detect neuropeptide Y, which is a different form of the neurotransmitter [76]. The microelectrodes were coated with carbon fiber and platinum. Through the conjugation between aptamer and neuropeptide Y, the change in impedance with neuropeptide Y concentration was measured. This technique allows the monitoring of neurotransmitter concentrations in real time without the need for labeling, thus enabling exceptional precision and fast reaction times [77]. For instance, the researchers demonstrated a novel, label-free biosensor for the detection of acetylcholine using a single enzyme (acetylcholinesterase) and EIS [78]. Acetylcholinesterase was covalently immobilized onto the surface of gold microelectrodes through an amine-reactive crosslinker dithiobis (succinimidyl propionate) (Figure 3d). Furthermore, they effectively passivated the gold electrode with SuperBlock, which eliminated or reduced any nonspecific response to other major interfering neurotransmitter molecules, such as dopamine, norepinephrine, and epinephrine. Acetylcholinesterase is an enzyme that rapidly breaks down acetylcholine into acetate and choline, increasing local H+ concentration. Acetylcholinesterase, immobilized on a gold electrode, forms an electrical double layer. As acetylcholine is degraded, ions at the enzyme surface change from one cation to two cations + one anion, altering local pH. With acetylcholinesterase degrading about 25,000 molecules per second, there is a significant impact on ion concentration, perturbing the double layer. This results in changes in capacitance and resistance, detectable via EIS. Using this strategy, the various concentrations of acetylcholine (from 5.5 μM to 550 μM) in whole blood could be sensitively detected using the fabricated label-free nanobiosensors without the need for any electrical or fluorescence tagging molecules. In another research study, an electrochemical biosensor was fabricated using a combination of multiwalled carbon nanotubes (MWCNTs) and nickel nanoparticles embedded in an anandamide-imprinted polymer [79]. The synthesized nickel nanoparticles were integrated with MWCNTs and molecularly imprinted polymer, resulting in a nanobiosensor with enhanced sensitivity and selectivity. Through the CV with K_3_Fe(CN)_6_/K_4_Fe(CN)_6_ solution, the current value of the bare electrode showed the lowest value (0.01 mA) among the electrodes at each fabrication step; however, after the fabrication steps, the fabricated nanobiosensor showed a maximum current response of 0.11 mA. The fabricated nanobiosensing platform demonstrated highly sensitive detection of anandamide, with a limit of detection of 0.01 nM and 50% stability over 4 months. Despite potential interfering components, such as acetylcholine and dopamine, owing to their chemical similarities, the fabricated sensor displayed selectivity toward anandamide. Furthermore, impedimetric nanobiosensors have been developed by researchers for the detection of dopamine. For instance, a novel electrochemical nanobiosensor based on zinc oxide with embedded polyvinyl alcohol nanoplatelets was developed for the determination of dopamine over a broad range, with a limit of detection of 5.0 nM [80]. In another case, a fullerene-pyrrole-pyrrole-3-carboxylic acid nanocomposite-modified molecularly imprinted impedimetric nanobiosensor was developed for the detection of dopamine in urine [81]. The study exhibited impressive sensitivity and enabled the detection of dopamine with a remarkable LOD value of 8.77 ng/mL.

### 3.4. Field Effect Transistor Biosensors

FET biosensors have emerged as prominent technologies in the field of biosensing owing to their high sensitivity and selectivity in detecting various biological substances, such as neurotransmitters [82]. The biosensors make use of the distinct electrical characteristics of semiconducting materials, thus enabling them to effectively transform biological interactions into quantifiable electrical signals [83]. The mechanism of FET biosensors is based on modulating the conductance of a semiconductor channel in response to the binding or interaction of the target neurotransmitter with a biorecognition element immobilized on the sensor surface [84]. In general, the gate of the FET biosensor is modified with a biomolecular receptor that is specific to the target neurotransmitter. This receptor can be an antibody, enzyme, or molecularly imprinted polymer, and its role is to selectively bind and capture the desired neurotransmitter [85]. When the neurotransmitter molecule forms a complex with the receptor protein located on the surface of the ion channel gate, it initiates a modification of the distribution of electrical charges, hence causing the electric potential difference across the semiconductor channel to be modified. The change in the surface charge leads to a variation in the conductance of the semiconductor channel, thus resulting in a measurable electrical signal. The FET biosensor’s capacity to convert molecular binding events into electrical modifications makes it a very promising framework for the rapid, label-free, and real-time identification of diverse analytes [86]. Consequently, it has attracted significant interest in the research of neurotransmitters. For instance, in one study, an electric-field-assisted assembly was employed to construct single-walled carbon nanotubes onto prepatterned electrodes by adopting an FET architecture [87]. By utilizing a mixture of micro- and nanofabrication methods complemented by microfluidics, the researchers effectively integrated the assembly into a highly advanced sensing platform. The combined setup enabled the monitoring of electrical signals in real time, and facilitated the reversible detection of several analytes. The drain-source current of the fabricated FET biosensor exhibited a significant decrease upon the introduction of dopamine, ascorbic acid, and adrenaline, ultimately stabilizing at a plateau after about 100 s. Furthermore, the optical detection of the analytes was conducted by measuring the fluorescence from single-walled carbon nanotubes in 96-well plates with the analytes. Through the incorporation of optical transduction, they demonstrated a correlation between an increase in fluorescence intensity and a decrease in the electrical current of single-walled carbon nanotubes for the detection of dopamine, epinephrine, and ascorbic acid. This approach allowed the simultaneous electrical and optical detection of these neurotransmitters and antioxidant compounds using the same sensing platform. These findings reveal the existence of common signal transduction mechanisms between optical and electrical sensors. While optical sensors offer noncontact readout advantages, thus making them suitable for in vivo applications, electrical sensors provide ease of on-chip integration. The correlation observed in the signal responses of both sensor types offers flexibility in choosing between the two readout technologies when designing next-generation nanobiosensors. In another study, the researchers developed a modular conductive metal–organic framework (c-MOF)-gated FET biosensor array for the sensitive detection of neurotransmitters utilizing two different types of c-MOF: Cu_3_HHTP_2_ (HTP) and Cu_2_TCPP (TCP) [88]. In this study, the c-MOF films served as a highly effective sensing layer, adsorbing analytes inside their pores. The c-MOF structures, functioning as a recognition module, exhibited the capacity to elicit distinct, nonspecific interactions with analytes determined by various factors, such as molecular size, charge, and binding affinity. The unique characteristic of fabricated nanobiosensors allowed the discrimination of signal transduction processes among analytes, without the need for either biological or complex synthetic receptors. As a result, the fabricated FET biosensor demonstrated high sensitivity, and was able to effectively perform the detection of catecholamines even in fluids with high ionic strength, thus resulting in clearly interpretable electrical signals. The limits of detection of the fabricated FET biosensor were 1.74 nM (HTP) and 1.95 nM (TCP) for dopamine, 1.52 nM (HTP) and 1.55 nM (TCP) for norepinephrine, and 1.66 nM (HTP) and 1.88 nM (TCP) for epinephrine. Moreover, acetylcholine was detected by employing a polyaniline-reduced graphene-oxide-based FET enzymatic nanobiosensor [89]. Owing to the doping effect in the graphene channel and polyaniline film for electrostatic enzyme immobilization, the sensitive electrochemical detection of the acetylcholine was achieved with an LOD of 72.3 nM.

## 4. Cell-Based Electrochemical Monitoring Systems for Neurotransmitters

As mentioned above, neurotransmitters play a critical role in signal transmission within the central and peripheral nervous systems. Deviations in neurotransmitter release can lead to significant neurological problems. In Section 3, various electrochemical sensors for the accurate and sensitive measurement of neurotransmitters outside the body, developed with the use of several bodily fluids, were presented and analyzed. However, measuring neurotransmitter concentrations based on bodily fluids can introduce potential error during sample collection and processing. To address this issue, researchers are developing electrochemical sensors that directly measure the neurotransmitters released from neuronal cells and neural stem cells. These electrochemical sensors utilize in vitro cell culture models to study neurotransmitter release dynamics and enable precise measurements. Additionally, implantable electrochemical sensors provide real-time monitoring of neurotransmitter levels, thus offering valuable insights into dynamic changes and their correlation with neurological disorders or treatment responses. The ongoing development of electrochemical sensors is promising for improving the accuracy and understanding of neurotransmitter dynamics in research and clinical applications.

### 4.1. In Vitro Neurotransmitter Detection System from Neuronal Cells Using Electrochemical Sensors

**Figure 4:**
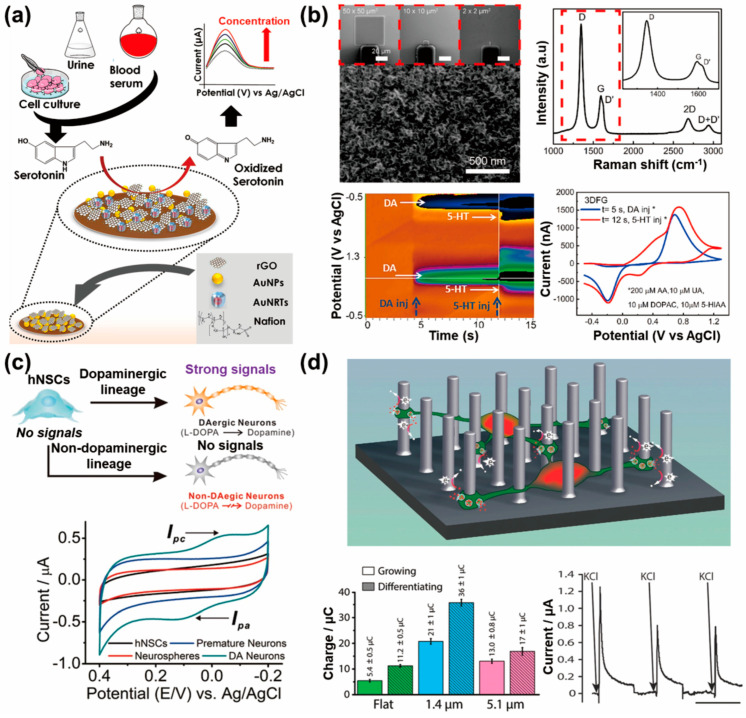
In vitro neurotransmitter detection system from neuronal cells and stem cells using electrochemical sensors: (**a**) Schematic of the sensor probe developed using Au-nanorattles (AuNRTs) and reduced graphene oxide (rGO) (AuNRTs-rGO) nanocomposite based on the serotonin detection mechanism (reprinted with permission from [90]; Copyright © 2022, Elsevier B.V.). (**b**) Scanning electron microscopy image and Raman spectra of three-dimensional fuzzy graphene microelectrode (**top**) and color plot (**bottom left**) and background-subtracted cyclic voltammogram showing reduction and oxidation peaks of injected dopamine and serotonin (reprinted with permission from [93]; Copyright © 2022 Elsevier B.V.). (**c**) Schematic representation of the conversion of hNSCs into dopaminergic (DAergic) and non-DAergic neurons (**top**) and cyclic voltammogram of cells undergoing differentiation into DAergic neurons (DA neurons) (**bottom**) (reprinted with permission from [17]; Copyright © 2015 WILEY-VCH Verlag GmbH & Co. KGaA, Weinheim). (**d**) Schematic of p3D-carbon where the cells differentiate at the bottom or between pillars (**top**), calculated average charge related to the amount of detected dopamine released by human neural stem cells (hNSCs) (**bottom left**), and characteristic current–time trace recorded during amperometric detection of dopamine (**bottom right**) (reprinted with permission from [102]; Copyright © 2014 WILEY-VCH Verlag GmbH & Co. KGaA, Weinheim).

Several neurotransmitters are nerve disorder diagnostic markers that can be used to conduct measurements in electrochemical detection systems in vitro. Of these, in vitro electrochemical biosensors for serotonin are being developed to address the need for precise measurements of the serotonin released from neurons. Serotonin plays a critical role in regulating mood and cognitive functions, and dysregulation is associated with neurological and psychiatric disorders. In vitro electrochemical biosensors allow the direct measurement of serotonin in controlled laboratory settings, thus providing high sensitivity and specificity. They enable real-time monitoring of serotonin dynamics from neuronal cells, offering insights into its temporal fluctuations and the severity of disorders. Mahato et al. developed a novel biosensor for performing fast detection of serotonin using nanocomposites of Au-nanorattles (AuNRTs) and rGO on gold nanoparticles (AuNPs) deposited on a glassy carbon electrode (Figure 4a) [90]. The biosensor exhibited a linear dynamic range of 3 × 10^−6^–1 × 10^−3^ M and a limit of detection of 3.87 (±0.02) × 10^−7^ M, thus making it suitable for normal and abnormal pathophysiological conditions. The sensor successfully detected serotonin in urine, blood serum, and in vitro models, thus demonstrating its direct clinical/practical applicability and long-term stability for up to 8 weeks. Nakatsuka et al. produced quartz nanopipettes with 10 nm orifices functionalized with aptamers that enabled the specific and selective recognition of serotonin [91]. These nanopipettes were able to detect physiologically relevant amounts of serotonin in complex environments like neurobasal media. The detection mechanism was investigated using complementary techniques such as quartz crystal microbalance and EIS, and a theoretical model supporting the experimental findings was proposed. This study highlighted the potential of aptamer-modified nanopipettes as rapid, label-free nanotools for diverse biological systems, thus offering high sensitivity and selectivity for the detection of small molecules. Ashraf et al. developed an ultrasensitive electrochemical sensor for the specific detection of serotonin in early disease diagnostics [92]. The sensor utilized a Cu_2_O metal oxide (MO)-incorporating CNT core, which was subsequently deposited with platinum nanoparticles (Pt NPs) on a CNTs-Cu_2_O-CuO nanopetal composite. The synergistic effects of improved surface area, conductivity, and electron transfer, induced by Pt NPs and the mixed-valence states of copper, enhnaced the electrocatalytic performance of the sensor, thus achieving a low limit of detection of 3 nM, a broad and linear concentration range, reproducibility, and durability. The proposed electrochemical sensor achieved the successful detection of serotonin in biotic fluids and real-time tracking of serotonin efflux from various cell lines, showcasing its potential as an early-disease diagnostic tool.

In addition to serotonin detection, the detection of dopamine using electrochemical methods is very promising in the fields of neuroscience and clinical diagnostics. As mentioned previously, dopamine is an important neurotransmitter that is involved in regulating various physiological processes, including mood, motivation, reward, and movement control. Abnormal dopamine levels are associated with neurological disorders, such as Parkinson’s disease, schizophrenia, and addiction. The electrochemical detection of dopamine makes it possible for researchers to study its release, uptake, and metabolism in real time, thus providing insights into its role in neurotransmission and disease pathology. Castagnola et al. implemented a high-sensitivity, real-time electrochemical sensor for multisite detection of dopamine in order to study its complex dynamics in the brain and improve treatments for neurological and neuropsychiatric disorders (Figure 4b) [93]. The presented three-dimensional fuzzy graphene (3DFG) microelectrode arrays demonstrated responses with exceptional selectivity, sensitivity (2.12 ± 0.05 nA/nM), and stability (stabilizing after at least 200 h, corresponding to 7.2 million cycles) for electrochemical sensing of dopamine. The high surface area of 3DFG allows miniaturization without compromising performance, thus making it a promising platform for high-resolution dopamine detection using fast-scan cyclic voltammetry (FSCV). Huang and co-workers developed a highly sensitive electrochemical sensor for dopamine detection using a composite of graphene quantum dots (GQDs) and MWCNTs [94]. The combination of GQDs and MWCNTs improved the conductivity of the electrodes and enhanced the selectivity for the purposes of dopamine detection. The sensor exhibited excellent performance with a broad linear range of 0.005–100.0 μM, a low limit of detection of 0.87 nM, and successful detection of dopamine in human serum and live PC12 cells. Yang et al. quantified the electrochemical performance of cavity carbon nanopipette electrodes (CNPEs) and applied them for dopamine detection using FSCV [95]. Cavity CNPEs exhibited fast temporal responses comparable to traditional carbon-fiber microelectrodes, while open-tube CNPEs had slow and varying temporal responses. The increased currents observed in cavity CNPEs were attributed to the trapping of small cavities. An increase in the local dopamine concentration resulted in an FSCV frequency-independent response and cyclization peaks typically observed with high concentrations of dopamine. Cavity CNPEs demonstrated higher selectivity for dopamine compared with ascorbic acid, and were able to detect dopamine in mouse brain slices without clogging, thus making them promising neurochemical sensors with nanoscale spatial resolution. Eom et al. presented an overoxidized polypyrrole/sodium dodecyl sulfate (SDS)-modified MWCNT electrode for highly sensitive and selective detection of dopamine [96]. The electrode prepared using SDS (as a dopant) and NaOH (as an oxidizing agent) enabled the detection of dopamine at concentrations as low as 5 nM, with a limit of detection of 136 pM. The electrode exhibited excellent selectivity for dopamine compared with interfering molecules such as ascorbic acid and glucose. Furthermore, the electrode demonstrated potential for performing the in vitro detection of dopamine secreted from dopaminergic cells (PC12 cells) and exhibited biocompatibility, thus making it a promising candidate for neural interface applications in monitoring dopamine concentrations in vitro and in vivo.

In terms of other neurotransmitters, the major excitatory and inhibitory neurotransmitters in the brain are glutamate and GABA, respectively. Doughty et al. developed a microwire-based electrochemical biosensor for measuring the real-time dynamics of glutamate and GABA in brain tissues [97]. The biosensors, coated with glutamate and reagent-free GABA, were designed in a geometrically improved plane for signal acquisition. The biosensors were able to successfully detect changes in glutamate and GABA levels in rat hippocampal slices and a freely moving rat over fourteen weeks, highlighting their potential for the study of neurotransmitter dynamics in various brain regions. Li et al. reported a graphene-based FET biosensor functionalized with synthesized glutamate receptors for real-time monitoring of glutamate release from cultured rat hippocampus neurons [98]. The immobilized metabotropic glutamate receptors (mGluR) on the graphene surface enabled the specific binding of target glutamate molecules, thus resulting in changes in charge density. The biosensor demonstrated high sensitivity, detected glutamate in the femtomolar range in cell culture medium, and enabled real-time monitoring of glutamate release from primary rat hippocampus neurons. The study proposed a novel approach for the specific detection of released glutamate molecules from differentiated neurons, enhancing our understanding of neuronal communication, and offered the possibility of detecting other electrochemically inactive small molecules released by cells. Scoggin et al. developed a highly sensitive enzymatic glutamate microbiosensor in the form of a platinum microelectrode array, enabling continuous and real-time measurements of glutamate concentration from multiple recording sites [99]. The microbiosensor exhibited nearly four-fold higher sensitivity to glutamate compared to previously reported enzymatic sensors. By analyzing glutamate dynamics in cultured astrocytes and glioma cells, the microbiosensor effectively distinguished normal versus impaired glutamate uptake, showcasing its potential for monitoring and understanding glutamate signaling in both normal and pathological conditions. Additionally, the microbiosensor can be utilized to evaluate the effects of therapeutic drugs in treating various neurological diseases.

### 4.2. Stem-Cell-Released Neurotransmitter Detection Systems Using Electrochemical Sensors

In recent years, attempts have been made to utilize stem cells for the treatment of various diseases. Neural cells pose challenges in terms of regeneration and functional restoration compared with other cell types. Therefore, the differentiation of stem cells into neural cells in vitro and their subsequent transplantation is actively being researched. However, assessing the accuracy of neural differentiation remains difficult, thus making transplantation-based therapies inherently risky. During the process of differentiation, stem cells release neurotransmitters, and precise and sensitive measurements of these neurotransmitters cannot not only facilitate the therapeutic use of stem cells, but also enhance our understanding of their properties. Accurate measurement of neurotransmitter release would provide valuable insights into the functionality of differentiated neural cells and contribute to the development of safer and more effective stem-cell-based therapies. Kim et al. fabricated a cell chip using indium tin oxide-coated glasses (ITO), gold nanoparticles (GNP), and RGD-MAP-C peptide composites to enhance electrochemical signals and the proliferation of human neural stem cells (HB1.F3) [100]. The composite electrode of ITO/60 nm GNP/RGD-MAP-C achieved the highest enhancement of voltammetric signals and cell proliferation. DPV revealed a negative correlation between cell viability and doxorubicin concentrations, showing the sensitivity of the cell chip for assessing the adverse effects of drugs on HB1.F3 cells, especially at low concentrations. The same research group developed large-scale homogeneous nanocup electrode arrays (LHONA) for the effective detection of dopamine production and for monitoring the differentiation of human neural stem cells (hNSCs) into dopaminergic neurons (Figure 4c) [17]. The LHONA platform demonstrated excellent performance at detecting dopamine at low and high concentrations, with an LOD of 100 nM. The nanoscale pattern sizes and nanotopographical characteristics of LHONA enhanced the functions of dopaminergic cells and allowed sensitive monitoring of dopamine production, thus making it a promising tool for the preclinical testing of dopaminergic neurons derived from stem cells and optimization of differentiation protocols. Furthermore, they developed a cylindrical gold nanoelectrode (CAuNE) platform using laser interference lithography and electrochemical deposition, with the CAuNE-700 nm electrode exhibiting the best performance for dopamine detection in a linear manner in the range of 1–100 µM and a limit of detection of 5.83 µM [101]. The homogeneous periodic features of CAuNEs allowed successful culturing of human neural cells and detection of dopamine in their presence, thus making the platform valuable for disease diagnosis, dopaminergic neuronal functional tests, and toxicity assessments on human neuronal cells. Amato et al. fabricated structurally patterned pyrolyzed three-dimensional carbon scaffolds (p3D-carbon) to differentiate hNSCs into dopamine-producing neurons and sense-released dopamine (Figure 4d) [102]. The p3D-carbon induced spontaneous differentiation of hNSCs into mature dopaminergic neurons with high efficiency and promoted neurite elongation. The conductive properties and 3D environment of p3D-carbon facilitated the electrochemical detection of a larger fraction of released dopamine compared with conventional two-dimensional (2D) electrodes, thus providing real-time confirmation of the fate of hNSC-derived neurons. This study introduced new conductive 3D scaffolds that enabled efficient hNSC differentiation and in situ monitoring of dopaminergic neuron development.

Research on differentiating stem cells into neurons is important for the development of treatments and understanding neurological disorders. However, traditional 2D cell culture models do not fully replicate the complexity of neural cells in the human body. In recent years, active research has been conducted on the development of neural cell models in the form of spheroids or organoids, aiming to more accurately emulate the CNS. Consequently, there are ongoing efforts focused on the development of techniques for analyzing neurotransmitters in real time from 3D neural cell models. Nasr et al. developed a novel method for creating functionalized borosilicate glass capillaries with nanostructured textures as electrochemical biosensors for detecting glutamate release from cerebral organoids derived from human embryonic stem cells [103]. The biosensor exhibited catalytic activity for glutamate oxidation with high sensitivity and a broad linear range from 5 µM to 0.5 mM, down to 5.6 ± 0.2 µM. It was able to successfully detect glutamate within the organoids at different time points, thus demonstrating its reliability and potential for the monitoring of glutamate levels over time in a culture system. Zanetti et al. developed a novel electrochemical sensing approach for detecting dopamine in human midbrain organoids that emulated the affected region in Parkinson’s disease [104]. Traditional analysis methods for these organoids are time consuming and invasive, and are not able to monitor organoid development. By employing a redox-cycling technique and modifying the sensor’s surface, the authors [104] achieved enhanced selectivity and sensitivity, and reduced interference. The developed approach was able to successfully detect and monitor dopamine release in both healthy and Parkinson’s-disease-specific organoids, confirming its potential for drug screening and personalized disease modeling applications.

### 4.3. In Vivo Neurotransmitter Monitoring Systems Using Electrochemical Sensors

Various diseases arise from the CNS that can severely compromise quality of life. Neurodegenerative disorders, such as Alzheimer’s disease and Parkinson’s disease, are prominent examples of brain disorders that lead to a decline in cognitive and motor abilities. These conditions are often accompanied by abnormal neurotransmitter secretion, either in insufficient or excessive amounts. The dysregulation of neurotransmitters contributes to the underlying pathophysiology of these diseases. Thus, real-time measurements of neurotransmitters within the brains of living organisms are extremely useful for understanding disease progression, the evaluation of treatment efficacy, and the facilitation of drug development. Accurate and precise monitoring of neurotransmitter dynamics can provide valuable insights into the functional state of the brain, thus providing a deeper understanding of the mechanisms underlying neurodegenerative disorders.

**Figure 5:**
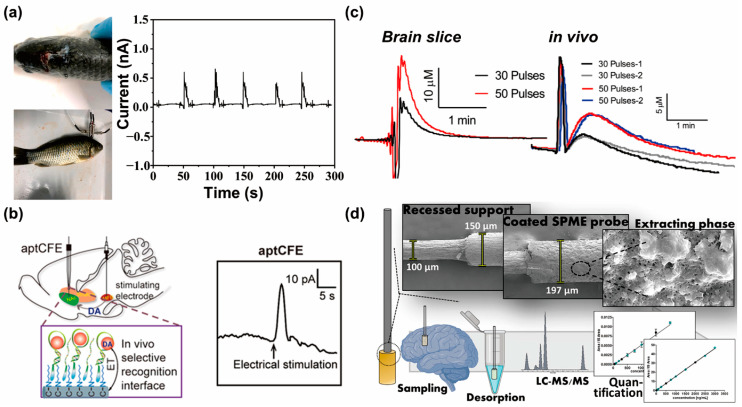
In vivo neurotransmitter monitoring system using electrochemical sensors: (**a**) photograph of the crucian fish brain, the field-effect transistor biosensor setup on the fish brain (**left**), and the I_ds_ output signal of the crucian fish brain monitored by the biosensor (**right**) (reprinted with permission from [105]; Copyright © 2021, Elsevier B.V.). (**b**) Schematic of aptCA-functionalized carbon fiber electrodes (CFEs) for in vivo dopamine sensing (**left**), and the current responses of aptCA-functionalized CFEs in the rat nucleus accumbens upon electrical stimulation of the rat’s medial forebrain bundle (**right**) (reprinted with permission from [109]; Copyright © 2020, Wiley-VCH GmbH). (**c**) Amperogram of stimulated glutamate release in the subthalamic nucleus of a rat brain slice (**left**) and stimulated rat brain in vivo (**right**) (reprinted with permission from [110]; Copyright © 2019, Elsevier B.V.). (**d**) Schematic of the solid-phase microextraction-based approach used for quantitative measurements of multiple neurotransmitters (reprinted with permission from [114]; Copyright © 2019, American Chemical Society).

In recent years, multiple research groups have expended considerable efforts in the development of techniques for the direct insertion of electrodes into the brain. These electrodes are designed to detect and measure neurotransmitter secretion in real time, with high sensitivity and temporal resolution. By monitoring neurotransmitter dynamics, researchers aim to unravel the intricate relationship between neurotransmitter imbalances and the progression of neurodegenerative diseases. In the case of in vivo dopamine measurements, Liu et al. developed a regenerated FET biosensor combined with an in vivo monitoring system for dopamine detection (Figure 5a) [105]. The biosensor utilized gold-coated magnetic nanoparticles as a recognition unit for dopamine and allowed simple regeneration following the removal of a permanent magnet. The FET biosensor demonstrated high sensitivity, selectivity, and stability, with a linear response in the range of 1–120 μmol L^−1^ and a low-limit of detection of 3.3 nmol L^−1^. This innovative platform enabled online and remote control of the sensitivity and the limit of detection, providing potential for reusability, cost effectivness, and large-scale production. Taylor et al. developed a PEDOT/CNT nanocomposite coating for highly sensitive and selective detection of resting dopamine (DA) [106]. The PEDOT/CNT-coated carbon fiber electrodes (CFEs) exhibited a significantly increased sensitivity for detecting resting DA. Implantation of PEDOT/CNT-functionalized CFEs in the rat dorsal striatum allowed the measurements of absolute basal DA concentrations and monitoring changes in DA levels in response to drug administration. Additionally, functionalizing PEDOT/CNT onto gold electrode sites in silicon microelectrode arrays enabled multisite DA sensing with excellent spatial resolution, thus offering a versatile approach for high-resolution tonic DA detection. Liu et al. reported a miniaturized microprobe system for wireless optical interrogation and neurochemical sensing in deep brain regions [107]. The system combined microlight-emitting diodes for optogenetic stimulation and PEDOT:PSS-coated diamond films for the electrochemical detection of dopamine. The implantable optoelectrochemical probes, controlled remotely and wirelessly, were successfully used for optogenetic interference and real-time detection of dopamine release in freely behaving mice. This integrated approach demonstrated the potential for simultaneous optical control and electrochemical sensing in complex nervous systems. Raju et al. implemented novel polymer and waveform modifications to enhance the detection of 3-methoxytyramine (3-MT), 3,4-dihydroxyphenylacetic acid (DOPAC), a dopamine metabolite neurotransmitter [108]. The application of a specific waveform for DOPAC detection improved its sensitivity by eliminating interference from the dopamine waveform. Additionally, the use of positively charged cationic polymers (like polyethyleneimine) enables the preconcentration of DOPAC on the electrode’s surface, further enhancing detection sensitivity. These findings have implications for the development of electrode materials and waveform strategies to detect important biomolecules, such as dopamine metabolites. Hou et al. presented a novel interfacial functionalization strategy using aptamer cholesterol amphiphiles (aptCAs) to immobilize aptamers onto alkyl chain-functionalized CFEs (Figure 5b) [109]. This approach enabled the development of highly selective systems for the investigation of neurochemical dynamics in living systems. The successful immobilization of aptamers on the CFE surface offers new possibilities for designing in vivo sensors to explore brain chemistry.

In addition to in vivo dopamine detection systems, other in vivo neurotransmitter sensing systems have also been developed using electrochemical techniques. Ganesana et al. developed an electrochemical biosensor with a diameter of 50 µm for real-time monitoring of L-glutamate in vivo (Figure 5c) [110]. The biosensor incorporated a permselective poly o-phenylenediamine glutamate oxidase membrane and ascorbate oxidase to enhance selectivity and eliminate interferences. The biosensor exhibited a linear response in the range of 5–150 µM, high sensitivity, rapid responses, and 1-week storage stability, thus making it suitable for future applications in glutamate detection. Castagnola et al. demonstrated the successful detection of exogenous melatonin in the brain using FSCV on pre-activated CFEs [111]. The CFEs exhibited high sensitivity and low limits of detection for melatonin detection, with improved stability and minimal fouling following prolonged preconditioning. The FSCV method enabled drift-free, long-term monitoring of administered melatonin in mouse brains, thus providing reliable and continuous measurements of melatonin concentration and dynamics. Additionally, electrochemical biosensors with integrated circuitry have recently been developed that are able to realize the sensitive in vivo detection of neurotransmitters. Since these miniaturized biosensors can achieve high sensing performance, including recording and signal processing by complex circuits in the biosensors, the circuit-integrated electrochemical biosensor has the potential to advance the in vivo monitoring of neurotransmitters. For instance, a wireless and battery-free implantable electrochemical biosensor was developed [112]. The developed device was simultaneously capable of optogenetic stimulation and the identification of electrochemical signals in vivo due to it being a miniaturized biosensing system with a complex circuit that included wireless power transfer technology. In another study, a glass carbon electrode with flexible circuits was utilized to detect dopamine in vivo [113]. The electrode was coated with PEDOT and carbon nanotubes to decrease the impedance of the electrode. Employing multiple channels on the electrode, in vivo phasic dopamine release was monitored.

Among the in vivo neurotransmitter sensing systems presented, in some studies, multiple neurotransmitters were successfully measured using a single electrode. Lendor et al. developed a solid-phase microextraction-based (SPME) approach as a minimally invasive method for performing quantitative measurements using multiple neurotransmitters in vivo, including dopamine (Figure 5d) [114]. The optimized miniaturized SPME probe enabled simultaneous sampling and sample preparation, with tailored coatings for the extraction of small hydrophilic molecules. The developed SPME-HPLC-MS/MS protocol demonstrated accurate and precise measurements of neurochemicals in a surrogate brain matrix, with potential applicability to the study of neurochemical levels in various brain systems implicated in psychiatric disorders. Li et al. developed a novel neurochemical biological interface called NeuroString, which consisted of a stretchable, tissue-mimicking material for sensing neurotransmitters [115]. The NeuroString sensors enabled long-term, real-time, and multiplexed monitoring of monoamine levels in the brains of freely behaving mice, and enabled serotonin dynamics measurements to be performed in the gut without disrupting natural movements. This flexible and conformable biosensing interface could potentially be used to study the effects of neurotransmitters on gut microbes and brain–gut communication, and has implications for biomolecular sensing in other soft organs in the body. Xie et al. reported an organic electrochemical transistor array (OECT-array) technique for the real-time monitoring of catecholamine neurotransmitters (CA-NTs) in rat brains [116]. The OECT array allowed the sensitive and rapid detection of nanomolar concentrations of CA-NTs with a low working voltage and long-term stability. By utilizing this technology, the complex interplay between different brain regions and pathways involved in dopamine release could be simultaneously mapped, thus providing insights into the reciprocal innervation between the ventral tegmental area and the substantia nigra pars compacta. Recent advancements in electrochemical techniques, made possible by the utilization of smaller electrodes, have ushered in new opportunities for the precise measurement of storage and regulatory processes within individual living cells [117,118]. One notable development in this field is the introduction of ‘intracellular vesicle impact electrochemical cytometry’ by Li et al., a technique that enables the quantification of catecholamines within individual vesicles [119]. This innovation has been widely adopted, and has played a pivotal role in enhancing our comprehension of the ‘partial release’ hypothesis, especially when juxtaposed with the quantities of catecholamine released through exocytosis. These advancements in real-time measurements of neurotransmitters offer a promising avenue for both clinical applications and basic research. They have the potential to revolutionize the field of neuroscience, thus providing crucial insights into the underlying neurochemical changes associated with neurological disorders. Furthermore, this technology introduces new possibilities for the development of targeted therapies and personalized treatment approaches tailored to individual patients based on their unique neurochemical profiles.

## 5. Conclusions and Future Perspectives

Neurotransmitters are important substances in the neurotransmission system, and various neurotransmitters are used for the transmission of signals among nerve cells. As such, when important neurotransmitters are absent or fail to fulfil their essential roles, they can cause cranial neurological diseases; this will invariably lead to the occurrence of other diseases, such as cancer. Therefore, accurate neurotransmitter sensing is essential for the early diagnosis and treatment of neurological and other diseases. To this end, numerous electrochemical biosensors have been developed thus far, and with the introduction of nanomaterials and nanotechnologies, nanobiosensors with increased sensitivity and the capability to conduct multiple diagnoses have been reported for the detection of neurotransmitters. Moreover, these nanobiosensors can not only be used to diagnose neurotransmitters, but can also serve as the basis for the development of neural stem cell therapeutics by monitoring neurotransmitters derived noninvasively during the neural stem cell differentiation process. In this review, interdisciplinary information about electrochemical nanobiosensors incorporating nanomaterials and nanotechnologies with the aim of achieving the accurate detection and monitoring of neurotransmitters was discussed. For this purpose, representative neurotransmitters were provided. Recently reported electrochemical nanobiosensors incorporating nanomaterials or nanotechnologies in order to detect these neurotransmitters were also discussed; these were categorized into (i) direct neurotransmitter detection action regardless of living cells, (ii) cell chips for disease-related neurotransmitter detection, and (iii) stem-cell-related neurotransmitter detection. The recently conducted studies in which the electrochemical neurotransmitter biosensors discussed in this review were developed are summarized in Table 2.

These electrochemical nanobiosensors are applicable to disease diagnosis, stem cell differentiation processes, and organoid production and theragnosis [120,121]. For instance, these nanobiosensors can be utilized to monitor the production of brain organoids composed of neurons, construction of the neurological disease model, and can verify organoid characteristics. Additionally, these nanobiosensors can serve as basic tools for the organ-on-a-chip development, as well as the development of new drugs [122]. In addition, they can be used for theragnostic applications of neurological diseases based on the combination of real-time diagnosis and medical treatments, which have recently been attracting attention in the biomedical field [123]. Furthermore, it has been reported that some neurotransmitters have further roles (beyond simple intrasynaptic signal transmission), including brain functioning [30,124]. Additionally, by focusing on its inherent function for monitoring, electrochemical sensors can be applied to clinical investigations, along with the flexible/wearable/implantable electronic devices that have recently begun to attract attention in the biosensor field, enabling the practical real-time monitoring of neurotransmitter-related diseases in patients [125,126,127]. Electrochemical nanobiosensors can also be used as a core component in the development of probe-free electronic devices capable of performing sensing functions such as the electronic nose/tongue, for the monitoring of various targets, including neurotransmitters [128]. Additionally, in the near future, by combining biosensing functions with the fields of machine learning and artificial intelligence, they are expected to play a role as an analysis tool not only for achieving precise and sophisticated diagnosis, but also for performing real-time data analysis [129]. In conclusion, the research on the development of electrochemical neurotransmitter nanobiosensors covered in this review is expected to have a tremendous biomedical impact, ranging from organoids to organs on a chip, and from theragnosis to future electronics.

**Table 2:** Representative electrochemical nanobiosensors used for the detection of neurotransmitters illustrated in this review.

Sensing Techniques	Targets	Nanomaterials/Nanotechniques	Sample Type	Limit of Detection	Reference
Amperometric	Dopamine	CDs-CPTMS	Cell-free	1.0 nM	[53]
	Catecholamine	Chitosan nanoparticle	Cell-free	0.17 µM	[54]
	Acetylcholine	Poly(3,4-ethylenedioxythiophene) (PEDOT)/carbon electrodes	Cell-free	Not applicable (N/A)	[55]
	Acetylcholine	Platinum nanoparticles/porous graphene oxide nano sheets	Cell-free	1 nM	[56]
	Serotonin	Pt NPs on the carbon nanotubes (CNTs)-Cu_2_O-CuO	HaCaT cell 4t1 cell P815 cell	3 nM	[92]
	Glutamateγ-aminobutyric acid	Microwire	Rat hippocampal slices	N/A	[97]
	Glutamate	Platinum microelectrode array	Astrocyte	6.3 µM	[99]
	Dopamine	Three-dimensional carbon scaffolds (p3D-carbon)	Human neural stem cells	N/A	[102]
	Glutamate	Borosilicate glass capillaries	Human cerebral organoids	5.6 µM	[103]
	Dopamine	Interdigitated gold microsensor	Human midbrain organoids	476 nM	[104]
	Dopamine	Carbon fiber electrode (CFE)	Rat nucleus accumbens	N/A	[109]
	Glutamate	Poly o-phenylenediamine (PPD) membrane/glutamate oxidase/ascorbate oxidase	Rat brain slices and in vivo	0.044 µM	[110]
Voltametric	Dopamine	Polypyrrole-derived carbon nanotube	Cell-free	0.2 µM	[63]
	Epinephrine	Carbon quantum dot/copper oxide nanocomplex	Cell-free	15.99 µM	[64]
	Dopamine	Reduced graphene oxide sheets	Cell-free	0.11 μM	[72]
	Dopamine, Adenine	Ni-polyacrylamide-molecular imprinted polymer (PAM-MIP) matrix	Cell-free	0.12 μM 0.15 μM	[73]
	Serotonin	Gold (Au)-nanorattles (AuNRTs)/reduced graphene oxide (rGO)/Au nanoparticles (AuNPs)	Neuronal cell	0.39 µM	[90]
	Dopamine	Graphene quantum dots (GQDs)/multiwalled carbon nanotubes (MWCNTs)	PC12 cell	0.87 nM	[94]
	Dopamine	Cavity carbon nanopipette electrodes (CNPEs)	Mouse-brain slices	56 nM	[95]
	Dopamine	Overoxidized polypyrrole/sodium dodecyl sulfate (OPPy/SDS)-CNT electrode	PC12 cell	136 pM	[96]
	Dopamine	Homogeneous nanocup electrode arrays	Human neural stem cells	100 nM	[17]
	Dopamine	Cylindrical gold nanoelectrode	Human neural stem cells	5.83 µM	[101]
	Dopamine	PEDOT/CNT-functionalized CFEs	Rat dorsal striatum	2.03 nM	[106]
	Dopamine	PEDOT: PSS-coated diamond films	Ventral tegmental area of the mouse	0.5 μM	[107]
	Melatonin	CFEs	Mice brain	20.02 nM	[111]
	Dopamine, Serotonin	Graphene-based biosensing neural interface	Mouse brain/colon	5.6 nM 3.5 nM	[115]
EIS	Acetylcholine	Gold microelectrodes	Cell-free	N/A	[78]
	Anandamide	Nickel nanoparticles/Imprinted polymer	Cell-free	0.01 nM	[79]
	Dopamine	Zinc oxide-embedded polyvinyl alcohol nanoplatelets	Cell-free	5.0 nM	[80]
	Dopamine	Fullerene-pyrrole-pyrrole-3-carboxylic acid nanocomposite	Cell-free	8.77 ng/mL	[81]
	Serotonin	Quartz nanopipettes	Neurobasal medium	N/A	[91]
FET	Dopamine, Epinephrine	Single-walled carbon nanotubes/microfluidics	Cell-free	N/A	[87]
	Dopamine, Epinephrine, Norepinephrine	Conductive metal–organic framework (c-MOF)-gated field-effect transistor (FET) arrays: Cu3HHTP2 (HTP) (i) and Cu2TCPP (TCP) types (ii)	Cell-free	1.74 nM (i) 1.95 nM (ii) 1.66 nM (i) 1.88 nM (ii) 1.52 nM (i) 1.55 nM (ii)	[88]
	Acetylcholine	Polyaniline/reduced graphene oxide	Cell-free	72.3 nM	[89]
	Glutamate	Graphene	Primary embryonic rat hippocampal neurons	1 fM	[98]
	Dopamine	Gold-coated magnetic nanoparticles	Fish brain	3.3 nM	[105]

## Figures and Tables

**Table 1:** Representative neurotransmitters with their major functions and locations.

Neurotransmitter	Function	Location
Acetylcholine	Muscle control Memory	Central nervous system Peripheral nervous system
Serotonin	Intestinal movement Mode regulation Sleep	Central nervous system Gut
Dopamine	Voluntary muscle movement Cognition Reward pathways	Hypothalamus
Norepinephrine	Fight/flight response	Adrenal medulla
GABA	Inhibits central nerve system	Brain
Glutamate	Excitatory neurotransmitter Memory	Central nervous system Peripheral nervous system

## Data Availability

Not applicable.
